# A composite score of serum cytokines enables early identification of patients at high risk for irAEs under immune checkpoint inhibition

**DOI:** 10.3389/fimmu.2025.1733357

**Published:** 2025-11-27

**Authors:** Nina Flatt, Antje Walter, Corinna Kochanek, Maximilian Beikirch, Viola K. DeTemple, Yenny Angela, Imke von Wasielewski, Ralf Gutzmer, Katrin Schaper-Gerhardt

**Affiliations:** 1 Department of Dermatology, Skin Cancer Center Minden, Johannes Wesling Medical Center Minden, Ruhr University Bochum, Minden, Germany; 2 Department of Dermatology and Allergy, Skin Cancer Center Hannover, Hannover Medical School, Hannover, Germany

**Keywords:** immune checkpoint inhibitors, immune related adverse events, cytokines, skin cancer, melanoma, biomarker

## Abstract

**Background:**

Immune checkpoint inhibitors (ICI) have revolutionized the treatment of advanced skin cancers. However, potentially severe and irreversible immune-related adverse events (irAEs) represent a major clinical challenge. Identifying reliable predictive biomarkers for irAEs remains critical to balancing efficacy and toxicity.

**Methods:**

In this prospective cohort of 131 skin cancer patients receiving ICI we analyzed the expression of 57 cytokines in baseline and longitudinal serum samples and tested their predictive value regarding the occurrence of irAEs.

**Results:**

We observed distinct cytokine expression profiles in patients who developed irAEs compared to those who did not, with variations reflecting the affected organ systems, particularly liver and thyroid toxicity. Elevated levels of IL-1RA and CXCL-13 and downregulated levels of IL-7 after the first ICI application significantly correlated with irAEs occurrence. To improve predictive accuracy, we developed a composite cytokine risk score integrating these cytokines, which independently predicted irAE in both univariable and multivariable models. ROC analysis of the cytokine risk score yielded an AUC of 0.710. Time-dependent Cox regression confirmed the cytokine risk score as an independent predictor of irAEs (univariable HR = 1.801 [95%CI=1.424–2.277; p<0.001]; multivariable HR = 1.407 [95%CI=1.072–1.846; p=0.014]). After stratifying patients into low- and high-risk groups, the high-risk patients had a significantly increased hazard of experiencing irAEs.

**Conclusion:**

IL-7, IL-1RA, and CXCL-13 represent potential biomarkers for irAEs risk stratification.

## 1 Introduction

The approval of immune-checkpoint inhibitors (ICI) has revolutionized the treatment of a variety of cancer entities. Especially in dermato-oncology, ICI have markedly improved long term overall und progression free survival in patients with metastatic melanoma and Merkel cell carcinoma (MCC) (1–3). In addition, the adjuvant use of ICI has been established for stage IIB, IIC and III melanoma (according to AJCC classification of 2017) (4), showing prolonged recurrence-free and distant metastasis-free survival in placebo-controlled trials (5). Promising results have also been reported for unresectable cutaneous squamous cell carcinoma (cSCC) and basal cell carcinoma (BCC) (6, 7).

By targeting inhibitory immune checkpoints such as cytotoxic T-lymphocyte-associated antigen 4 (CTLA-4) and programmed cell death protein 1 (PD-1) or its ligand (PD-L1), ICIs enhance T-cell activation and proliferation (8). This immune reactivation restores effective antitumor immunity but simultaneously disrupts self-tolerance, which can result in a broad spectrum of immune-related adverse events (irAEs).

These irAEs represent a major clinical challenge, as they may affect any organ system similar to classical autoimmune diseases. The risk and severity of these adverse events are notably increased with combined ICI, often leading to the development of multiple or high-grade toxicities (9). Importantly, severe irAEs can lead to treatment interruption or discontinuation, irreversible organ damage associated with a significant reduction in quality of life or even death. These risks are particularly relevant in the context of increasing ICI use in earlier disease stages, where the risk-benefit ratio may differ from that in metastatic settings (10). Consequently, the identification of predictive biomarkers for irAE development is crucial to enable early risk stratification, individualized monitoring, and improved management strategies to ensure both treatment efficacy and patient safety.

In this study we aimed to identify biomarkers that are easily available and can be integrated into routine diagnostics. We prospectively studied the concentration of different cytokines and chemokines in the serum of skin cancer patients who underwent immunotherapy. Baseline values as well as dynamic changes after the first ICI application were correlated with the occurrence of irAEs, leading to a predictive score.

## 2 Patients and methods

### 2.1 Study design and patient cohort

In this prospective study, we included 131 patients diagnosed with skin cancer (malignant melanoma, cutaneous squamous cell carcinoma, Merkel cell carcinoma), who were treated at the Department of Dermatology, Medical School Hannover from April 2019 to May 2023. All patients were in advanced stages and received either PD-1 monotherapy or a combined checkpoint inhibition including PD-1 and CTLA-4 inhibitors. Longitudinal serum samples were collected per patient at two different time points: pre-treatment (baseline) and 3–4 weeks after the first treatment cycle. The study design is shown in **Supplementary Figure S1**. All serum samples were centrifuged at 3000g for 15 minutes and supernatants were stored at -80°C until further analyses. Patients were monitored regularly over the course of therapy to allow early detection of irAEs. If irAEs occurred, affected organ, type of irAEs, severity, onset time and treatment regimen were thoroughly documented according to the National Cancer Institute**’**s Common Terminology Criteria for Adverse Events, version 4.0. (CTCAE).

### 2.2 Cytokine determination

Serum samples were analyzed using customized highly sensitive electrochemiluminescence immunoassays on the MESO Quickplex SQ120 according to the manufacturer**’**s instructions (V-Plex 54, U-Plex) (Mesoscale Discovery, Maryland, USA). Data were imported into the Mesoscale Discovery Workbench 4.0 analysis software for quantification (Mesoscale Discovery, Maryland, USA). Standard curves were used to assess the correct concentrations. If concentrations were lower than the detection limit, the parameter was excluded. Additionally, we collected routine laboratory parameters (LDH, white blood cell count, neutrophil counts, eosinophil counts, lymphocyte counts). The study was approved by the ethics committee in Hannover in accordance with the Declaration of Helsinki (vote: Nr 8685_BO_K_2019). Written informed consent of the patients was obtained.

### 2.3 Statistical analysis

For statistical analysis and visualization of the results GraphPadPrism (version 9.02), SPSS (version 29.0) and R (version 4.5.0.) was used. Illustrations were made in BioRender. Differences in the Log2FC with regard to cytokine concentrations between baseline and 3–4 weeks after therapy initiation were evaluated with the Wilcoxon-signed rank test. Differences between stratified patient groups and clinical characteristics were evaluated using the Chi^2^ Test. In order to evaluate the predictive potential of specific parameters, univariable and multivariable analyses were carried out by the logistic regression model and the Cox proportional hazard model. A p-value less than 0.05 was regarded as statistically significant. To calculate the cytokine risk score, the values (the intercept and the respective regression coefficients) obtained from a multivariable logistic regression analysis were subsequently used based on this formula: cytokine risk score = α + ß_1_*X_1_ + ß_2_*X_2_ + ß_3_*X_3_

α = intercept; ß_1,_ ß_2,_ ß_3_ = regression coefficients, X_1,_ X_2,_ X_3=_ cytokine values.

## 3 Results

### 3.1 Patient characteristics

From April 2019 to May 2023, we enrolled a total of 131 consecutive patients, who received ICI as a standard of care at Hannover Medical School. The median follow-up time from start of ICI was 18 months (inter quartile range: 11–27 months). The median age was 65 years and 55.7% of the patients were male. Melanoma was the most frequent cancer entity (n=122) followed by cSCC, MCC and mucosal or uveal melanoma. Half of the patients were categorized as stage III (49.6%) and the other half as stage IV (50.4%) according to AJCC 2017. Most patients received anti-PD-(L)1 monotherapy (mICI) (76.3%) whereas a minority was treated with a combined ICI (cICI) of anti-PD-1 and anti-CTLA-4 therapy (23.7%). 43.5% of the patients carried a BRAF mutation. The patient characteristics are summarized in **Table 1**.

**Table 1:** Patient characteristics.

Parameters		Total cohort	irAE		*p-*Value*
			no	yes	
		n (%)	n (%)	n (%)	
Patients		131	48	83	
Sex	female	58 (44.3)	23 (47.9)	35 (42.2)	0.523
	male	73 (55.7)	25 (52.1)	48 (57.8)	
ECOG	0	121 (92.4)	44 (91.7)	77 (92.8)	0.819
	1	10 (7.6)	4 (8.3)	6 (7.2)	
Age (years)	Median (min–max)	65 (31 – 90)	69 (34-90)	63 (31-89)	0.055
	years. Age > 65		28 (58.3)	34 (40.9)	
Disease	Melanoma	122 (93.1)	45 (93.7)	77 (92.8)	0.405
	SCC	2 (1.5)	1 (2.1)	1 (1.2)	
	MCC	1 (0.8)	1 (2.1)	0	
	Other	6 (4.6)	1 (2.1)	5 (6)	
Stage	III	65 (49.6)	23 (47.9)	42 (50.6)	0.767
	IV	66 (50.4)	25 (52.1)	41 (49.4)	
Therapy	mICI	100 (76.3)	45 (93.8)	55 (66.3)	<.001
	cICI	31 (23.7)	3 (6.2)	28 (32.7)	
Braf mutation	Yes	63 (48.1)	19 (39.6)	38 (45.8)	0.471
	No	57 (43.5)	25 (52.1)	38 (45.8)	
	Unknown	11 (8.4)	4 (8.3)	7 (8.4)	
Best response	CR	10 (15.9)	1 (4.5)	9 (22.0)	0.234
(Palliative Setting)	PR	9 (14.3)	3 (13.6)	6 (14.6)	
N=63	SD	13 (20.6)	4 (18.2)	9 (22.0)	
	PD	31 (49.2)	14 (63.6)	17 (41.5)	
Recurrence	No relapse	58 (85.3)	21 (80.8)	37 (88.1)	0.407
(Adjuvant Setting)	Recurrence	10 (14.7)	5 (19.2)	5 (11.9)	
N= 68					
Duration of FU	months (min-max)	18 (0-41)	19 (0-41)	13 (1-38)	

*Chi2-test.

mICI, monotherapy immune checkpoint inhibition, PD-1 and PD-L1 Antibody; cICI, combined immune checkpoint inhibition, (PD-1+CTLA-4); PFS, Progression free survival; OS, Overall survival; CR, complete response; PR, partial response; SD, stable disease; PD, progressive disease.

### 3.2 Occurrence of irAEs

**Figure 1:**
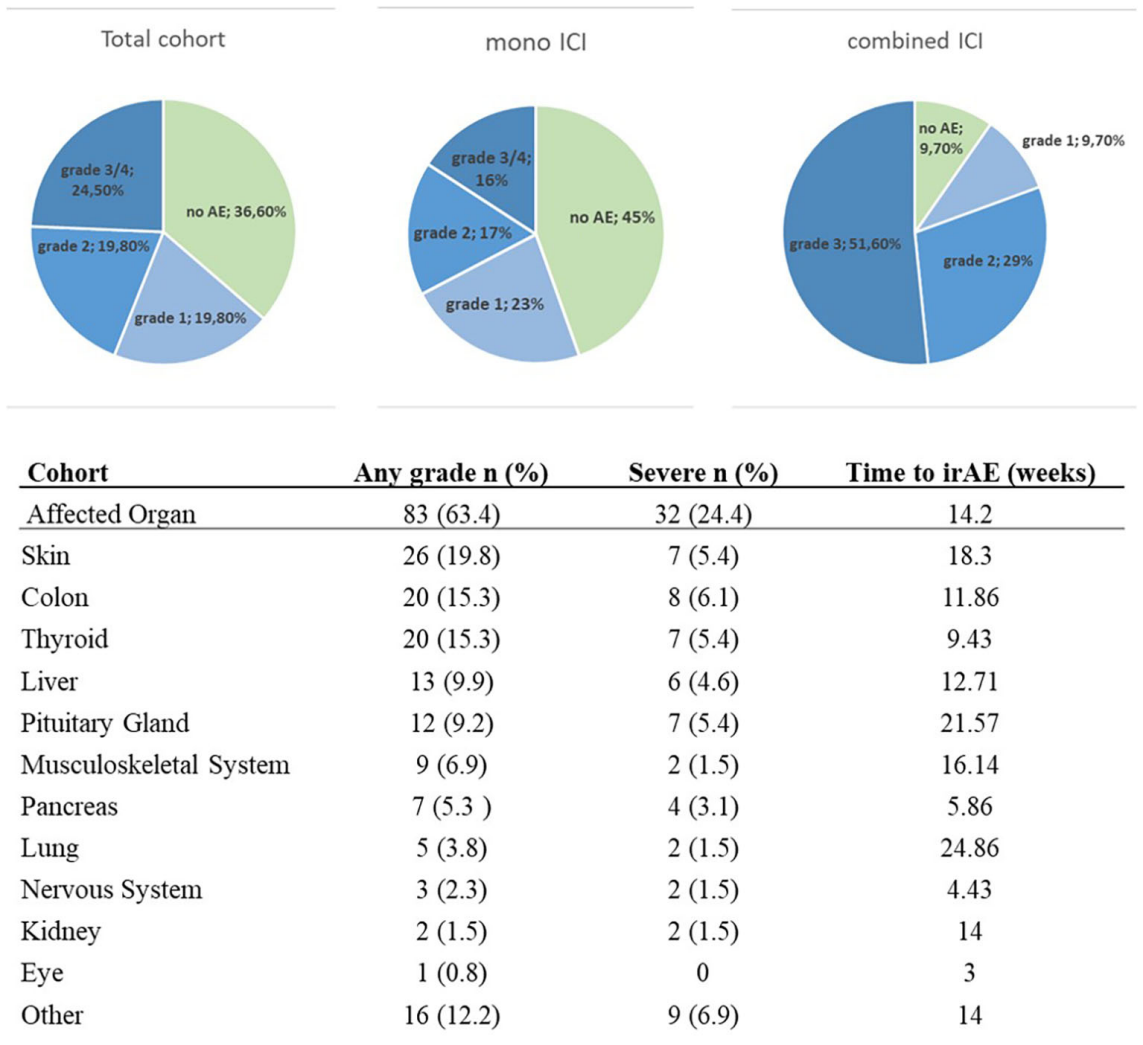
Distribution of irAE by severity grade, shown for the total cohort, for patients receiving PD-1 inhibition (mono ICI), and for patients treated with combined anti–PD-1 and anti–CTLA-4 therapy (combined ICI). The lower panel shows the distribution of organ-specific adverse events for any grade (grade 1-4) and severe grade (grade 3-4) as well as the median time period until the occurrence of irAE in weeks.

63.4% of the patients developed irAEs. Severe irAEs (CTCAE grade 3 and 4) occurred in 24.4% of the patients. The distribution of irAEs grades in regard of treatment regime can be found in **Figure 1**. The average time from therapy start to first irAEs symptoms was 14,2 weeks (**Figure 1**). The most frequently observed irAEs affected the skin (19.8%), colon (15.3%) and thyroid (15.3%). Among the key baseline patient characteristics, the use of combined ICI (cICI) with Nivolumab plus Ipilimumab significantly correlated with the occurrence of irAEs (**Table 1**). The other clinicopathologic variables sex, age, LDH and BRAF mutation showed no significant differences between patients with and without irAEs (Chi^2^-test, **Table 1**). We further analyzed baseline laboratory parameters (leukocyte, lymphocyte, eosinophil, and neutrophil counts, as well as the neutrophil-to-lymphocyte ratio [NLR] and derived neutrophil-to-lymphocyte ratio [dNLR]) prior to initiation of immunotherapy. We compared these parameters between patients who did not develop any irAEs and those who developed irAEs of any grade, or grade 3 or higher, or those with the most frequently observed irAEs (skin, colon, thyroid, pituitary gland, and liver).

Applying a Mann-Whitney-Test we did not detect any differences in baseline laboratory parameters between patients with and without irAEs. However, we observed a higher leukocyte count in patients with skin-related irAEs and a lower eosinophil count in patients with thyroid-related irAEs (**Supplementary Figure S2**).

### 3.3 Early changes in circulating cytokines predict irAEs

**Figure 2:**
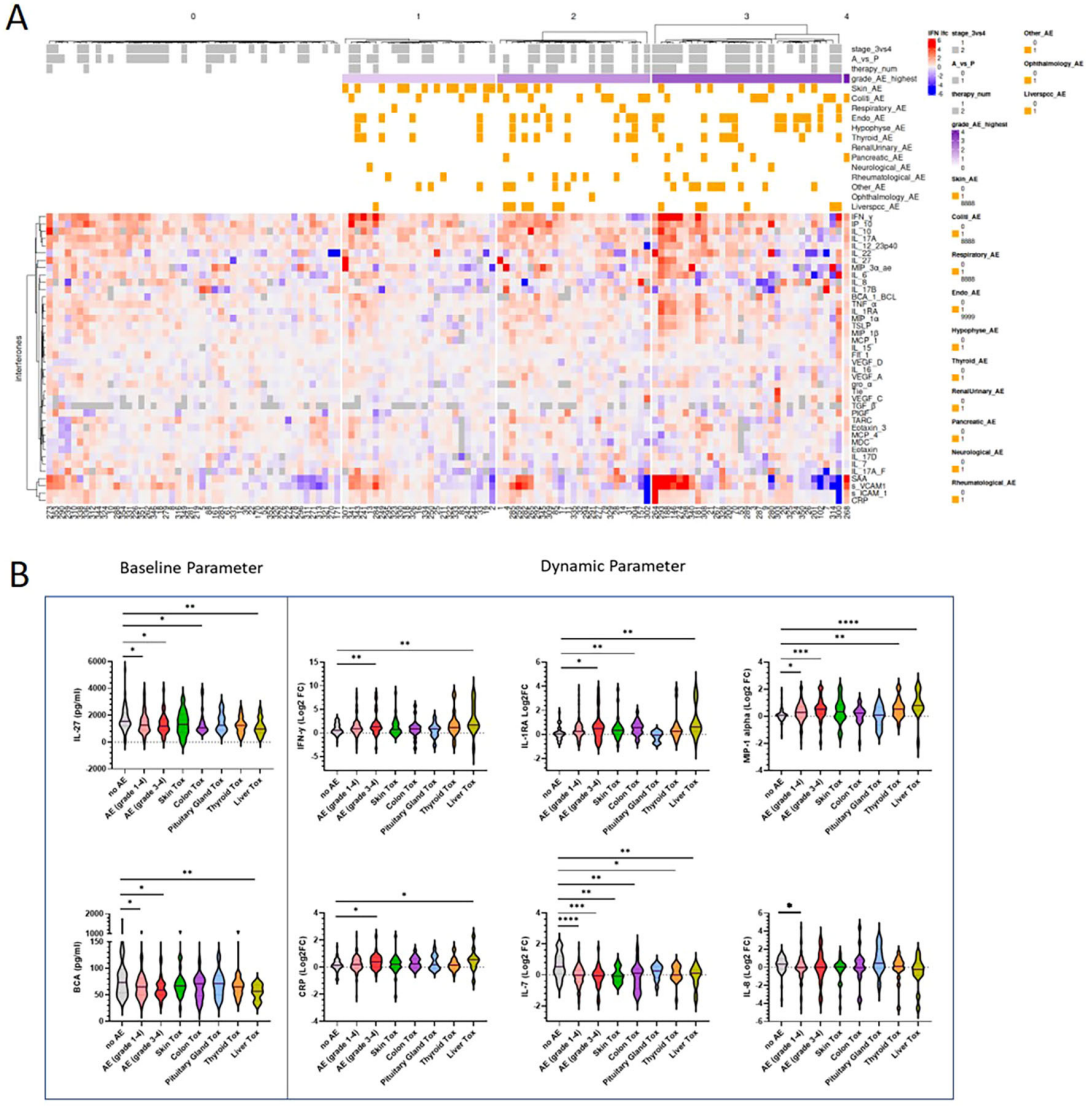
Early treatment changes in distinct serum cytokines are associated with irAEs. **(A)** Heatmap showing early treatment log2 fold changes (baseline to 3–4 weeks after first ICI administration) of 41 cytokines grouped by severity of irAEs (CTCAE grades 0-4). Patients were stratified based on the severity of immune-related adverse events (irAEs) into five distinct subgroups, per subgroup cytokines were subsequently clustered hierarchically. The top annotation of the heatmap illustrates clinical and therapy-related variables, including therapy type, disease stage, and the presence and grade of organ-specific irAEs. **(B)** The lower panel displays the cytokine expression profiles (log2 fold change). Violin plots showing log2 transformed cytokine fold changes between patients who developed classified irAE compared to patients with no irAE. Comparisons between groups were performed using Mann Withney U test. *p < 0.05, **p < 0.01, ***p <0.001; AE= adverse event, Tox= toxicity. .

In our cohort of 131 patients, we analyzed 57 different serum cytokines using electrochemiluminescence both at baseline as well as 3–4 weeks after the first ICI administration. Sixteen parameters were below the quantification limit and were therefore excluded from further analysis. Baseline concentration of the remaining 41 quantifiable parameters (listed in the heatmap in **Figure 2**) were assessed to determine whether cytokine expression could predict immune related toxicity. Therefore, we compared patients who developed irAEs of any grade to patients who did not experience any irAE at all during the observation period. In addition, we compared cytokine levels between patients who developed severe (CTCAE grade 3-4) irAEs and patients who showed no irAEs. From all tested baseline parameters only IL-27 and BCA/CXCL-13 showed a higher concentration in patients, who did not develop irAEs compared to patients with irAEs (**Figure 2B**).

To account for the baseline variability in cytokine concentrations and to assess early dynamic changes, we further studied relative cytokine changes and calculated the fold change (FC) in cytokine levels (post-treatment relative to pre-treatment). To visualize the dynamic cytokine pattern across the patient cohort, we performed hierarchical clustering based on longitudinal log2 FC in cytokines levels. Patients were stratified based on the severity of irAEs (0–4). Results are displayed in a heatmap (**Figure 2A**). We showed that patients with irAEs grade 3–4 display a strong upregulation of pro-inflammatory and interferon-related cytokines such as IFN-γ, IP-10, IL-6, and IL-1β. In contrast, patients without irAEs exhibited lower cytokine activity across the panel. These findings suggest that increasing irAEs severity is associated with a progressive shift towards a pro-inflammatory cytokine milieu. In **Figure 2B** cytokines are shown which are significantly upregulated on early treatment course in patients who developed irAEs (IFN-γ, IL-1RA, MIP-1alpha, CRP). However, some cytokines were rather downregulated in patients who experienced irAEs compared to patients without irAEs, in particular IL-7 and IL-8. This suggests that both activation and suppression of specific immune pathways may contribute to toxicity profiles.

Furthermore, the five most frequently affected organ systems were analyzed separately within the dataset, yielding distinct cytokine patterns. The most pronounced differences were observed between patients without irAEs and those who experienced adverse events in liver or thyroid, suggesting organ-specific cytokine signatures (**Figure 2B**).

In order to find early predictors for the occurrence of irAEs, we applied univariable logistic regression analysis with clinical as well as cytokine parameters. As expected, patients receiving cICI therapy exhibited a markedly higher incidence of irAEs (OR = 7,636 [95%CI=2,179-26,764; p<0,001]). In addition to treatment type, we identified further contributing factors such as patient age (OR = 0,971[95%CI=0,946-0,996; p=0,024]) and early changes in cytokine levels, specifically for IL-7 (OR = 0,381 [95%CI=0,193-0,755; p=0,006]), BCA-1/CXCL13 (OR = 2,438 [95%CI=1,173-5,069; p=0,017]), and IL-1RA (OR = 1,901 [95%CI=1,038-3,480; p=0,037]), following the first ICI administration. However, in multivariable analysis, only the type of therapy (p<0,03) and IL-7Log2FC (p<0,017) remained statistically significant (**Table 2**). The development of severe irAEs was further significantly associated with an increase of IFN-γ, Eotaxin-3, IL-27 and TSLP post treatment (**Supplementary Table S1**).

**Table 2:** Univariable and multivariable logistic regression analysis of patients with and without irAE (parameters above the line were included in multivariate regression) of different baseline and dynamic parameters.

Parameter	N	Univariable (p; OR 95% CI)	*p-value*	Multivariable (p; OR 95% CI)	*p-value*
Therapy (Ref.:mICI)	131	7,636 (2,179-26,764)	0,001	4,583 (1,115-18,178)	0,03
IL-7 (Log2FC)	130	0,381 (0,193-0,755)	0,006	0,407 (0,195-0,850)	0,017
BCA (Log2FC)	119	2,438 (1,173-5,069)	0,017	2,029 (0,781-5,268)	0,146
Age	131	0,971 (0,946-0,996)	0,024	0,990 (0,959-1,021)	0,521
IL1RA (Log2FC)	130	1,901 (1,038-3,480)	0,037	1,053 (0,495-2,237)	0,894
IL-27	130	1,000 (0,999-1,000)	0,074		
IFNy (Log2FC)	130	1,121 (0,962-1,526)	0,103		
IL-8 (Log2FC)	120	0,802 (0,581-1,108)	0,181		
IL-22 (Log2FC)	129	1,177 (0,960-1,443)	0, 117		
Sex (Ref.: male)	131	0,524 (0,388-1,619)	0,524		
CRP (Log2FC)	130	0,985 (0,904-1,074)	0,732		

OR, odds ratio; CI, confidence interval; FC, Foldchange; mICI, monotherapy immune checkpoint inhibition; irAE, immune related adverse events.

To enhance the predictive performance, we calculated a composite cytokine risk score for the occurrence of irAEs including cytokines with a significant p-value in univariable logistic regression model (IL-7Log2FC, BCALog2FC, IL-1RALog2FC). To calculate the cytokine risk score, we used the intercept and the respective regression coefficients of the cytokines derived from the multivariable logistic regression model. Subsequently, a ROC analysis was performed (AUC: 0.710) to determine the optimal cut-off of the cytokine risk score for stratifying the cohort into low- and high-risk groups, based on the Youden Index. The cytokine risk score was further evaluated in a time-dependent Cox regression model and emerged together with the cICI as an independent predictor of outcome in both univariable (HR = 1,801 [95%CI=1,424-2,277; p<0,001]) and multivariable analyses (HR = 1,407 [95%CI=1,072-1,846; p<0,014]) (**Table 3**). In order to validate the cytokine risk score and check for possible improvement, different methods of preprocessing, filtering or scaling were used. Detailed information can be found in supplements (**Supplementary Figure S3** and **S4**).

**Table 3:** Univariable and multivariable cox proportional hazard analysis of parameters in predicting irAE (grade1-4) following ICI in skin cancer patients.

Baseline/Dynamic Parameter	N	Univariable (p; HR 95% CI)	*p-value*	Multivariable (p; HR 95% CI) *p-value*	
Therapy regimen (Ref.mICI)	129	3,835 (2,392-6,147)	<0,001	2,463 (1,283-4,727)	0,007
Age	129	0,977 (0,963-0,992)	0,002	0,994 (0,976-1,012)	0,503
Cytokine risk score	117	1,801 (1,424-2,277)	<0,001	1,407 (1,072-1,846)	0,014
BCA (Log2FC)	117	1,974 (1,401-2,780)	<0,001		
IL1RA (Log2FC)	128	1,686 (1,244-2,283)	<0,001		
IL7R (Log2FC)	128	0,581 (0,403-0,838)	0,004		

HR, hazard ratio; CI, confidence interval; FC, Foldchange; mICI, monotherapy immune checkpoint inhibition; irAE, immune related adverse events.

**Figure 3:**
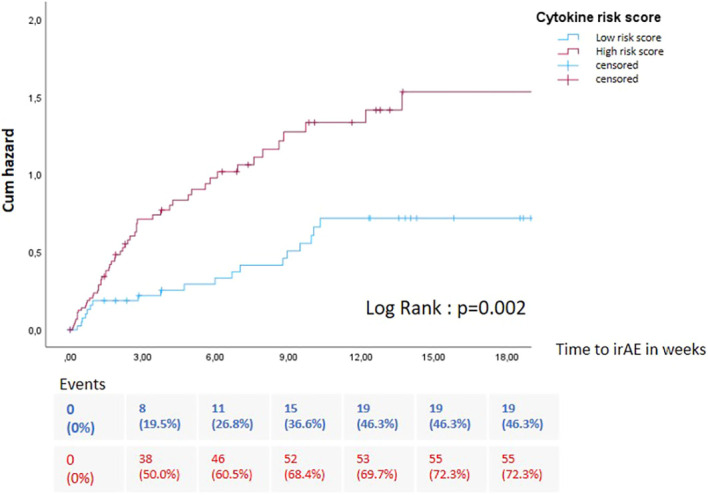
Kaplan–Meier analysis of the time-dependent occurrence of irAE stratified by cytokine risk score (high: red versus low: blue). Patients with a high cytokine risk score showed a significantly higher probability of developing adverse events (log-rank test, p = 0.002).

Stratification of the cohort according to the cytokine risk score revealed that patients with a high score were significantly associated with an increased hazard of experiencing irAEs (**Figure 3**). Moreover, further subgroup analysis indicated that patients with a higher cytokine risk score had a significant higher risk of irAEs affecting the skin, colon, thyroid and liver (**Supplementary Table S2**).

## 4 Discussion

The introduction of immune checkpoint inhibitors (ICIs) has significantly improved survival outcomes for many cancer patients, especially for melanoma. However, a substantial proportion of patients do not benefit from therapy but still exhibit the risk of developing irAEs. With the increasing use of ICI not only in the palliative setting but also in adjuvant and neoadjuvant settings (11–13), the identification of predictive biomarkers for the development of irAEs is of particular importance in order to guide treatment decisions and allow for a more personalized therapy application.

In the present study on skin cancer patients, we showed that combined ICI therapy is associated with a significantly higher incidence of irAEs, confirming previous findings across multiple tumor entities, including melanoma and cutaneous squamous cell carcinoma (14). However, adjusting for therapy we also identified individual cytokines, in particular IL-7, IL-1RA, and BCA/CXCL13, as predictive biomarkers for irAE development. Moreover, we integrated these cytokines in a composite cytokine risk score which demonstrated superior prediction of the occurrence of irAEs relative to single marker analysis. Importantly, also in subgroup analyses regarding severe irAEs and irAEs affecting specific organ systems the cytokine risk score remains predictive. This is of particular interest, since we could show that cytokine signatures appeared to differ depending on the organ system affected, with the most pronounced differences detected between patients without irAEs and those who experienced liver or thyroid toxicity.

IL-7 is a homeostatic cytokine essential for T cell survival and expansion; thus, a diminished IL-7 response in patients with irAEs may reflect an impaired immune regulation or an excessive peripheral consumption in the context of immune activation (15). Similarly, IL-1RA, a natural antagonist of IL-1, may reflect a counter-regulatory response to inflammation. CXCL-13 (BCA-1), known for its role in B cell chemotaxis and tertiary lymphoid structure formation, has also been implicated in autoimmunity and immunotherapy response (16).

Our findings align with other studies that have investigated cytokines as predictive biomarkers and key regulators of irAE in ICI-treated patients. We observed a correlation between higher severity of adverse events in our patients and a greater increase in pro-inflammatory Th1-cytokines such as e.g. IFN-γ and CXCL-10, which is in line with other reports (17–19). Furthermore, IL-1RA has been identified as a component of the Cytox-score proposed by Lim et al., which demonstrated predictive capacity for irAEs under ICI (20). Interestingly, the increased risk of irAEs in patients showing a post-treatment decline in IL-7 is supported by the results of Taylor et al. and Acar et al., who identified a germline IL-7 SNP associated with elevated IL-7 expression in B cells and a higher risk of toxicity under ICI (21, 22). While Taylor et al. point to a genetic predisposition reflecting a pre-activated lymphoid compartment, our data highlight that dynamic changes in IL-7 serum levels during therapy also carry prognostic value. A marked decrease in IL-7 may indicate utilization or dysregulation of IL-7–dependent T- and B-cell populations during immune activation, thereby contributing to autoimmunity. Together, these observations emphasize the central role of IL-7 in lymphocyte homeostasis and suggest that close longitudinal IL-7 monitoring could provide relevant information beyond static genetic markers for irAEs risk stratification and early detection.

Both IL-7 and CXCL-13, included in our cytokine risk score, are cytokines with key roles in B-cell biology (23, 24). IL-7 is critical for B-cell development and survival, while CXCL-13 directs B-cell trafficking to germinal centers and supports humoral immune responses. Their combined dysregulation in patients who develop irAEs may also reflect alterations in B-cell homeostasis or activation during ICI therapy, suggesting that B-cell–related pathways contribute to the pathogenesis of immune toxicity. In line with this, we also reported a case of a woman with melanoma and concomitant granulomatosis with polyangiitis who was treated with a B-cell–depleting therapy (Rituximab) and subsequently received ICI. Notably, B-cell depletion did not impair the efficacy of the CPI, and the patient did not experience any immune-related adverse events (25).

However, despite their promising approaches, the use of cytokines as predictive biomarkers for irAEs comes with several challenges. Cytokine levels can vary substantially between individuals due to genetic background, age, comorbidities, or prior therapies, which may confound their predictive value. Technical variability in cytokine measurement, including differences in assay platforms, sample handling, and batch effects, can further complicate data interpretation. Additionally, cytokine expression is highly dynamic and can be influenced by external factors such as infections, stress, or concurrent medications, making it difficult to distinguish changes specifically attributable to ICI therapy. Therefore, while cytokines hold translational potential, their use as biomarkers requires careful standardization and may be most effective when expressed either as relative values, integrated into composite scores or combined with clinical parameters, or as dynamic values during ICI therapy compared to an individual baseline at treatment initiation.

Regarding further clinical parameters, we also analyzed routinely generated blood parameters like leukocyte count, eosinophils or NLR. However, no parameters were associated with toxicity in line with findings in other studies (26).

The study’s limitations include its monocentric setting which introduces potential biases related to patient demographics, treatment protocols, and cytokine assay methodologies. Furthermore, the relatively small sample size, especially in subgroup analysis, restricts the statistical power and generalizability of our results.

## 5 Conclusion

In summary, our findings underscore the potential of IL-7, IL-1RA, and CXCL-13 not only as mechanistically informative cytokines but also as a combined biomarker score for risk stratification for the occurrence of irAEs in skin cancer patients. Future studies should validate these biomarkers prospectively in larger cohorts, ideally in conjunction with tissue-based immune profiling and genetic markers.

## Data Availability

The data that support the findings of this study are available from the corresponding author upon reasonable request.

## References

[1] Long GV Carlino MS McNeil C Ribas A Gaudy-Marqueste C Schachter J . Pembrolizumab versus ipilimumab for advanced melanoma: 10-year follow-up of the phase III KEYNOTE-006 study. Ann Oncol. (2024) 35:1191–9. doi: 10.1016/j.annonc.2024.08.2330, PMID: 39306585

[2] Wolchok JD Chiarion-Sileni V Gonzalez R Grob JJ Rutkowski P Lao CD . Long-term outcomes with nivolumab plus ipilimumab or nivolumab alone versus ipilimumab in patients with advanced melanoma. J Clin Oncol. (2022) 40:127–37. doi: 10.1200/JCO.21.02229, PMID: 34818112 PMC8718224

[3] D'Angelo SP Lebbé C Mortier L Brohl AS Fazio N Grob JJ . First-line avelumab treatment in patients with metastatic Merkel cell carcinoma: 4-year follow-up from part B of the JAVELIN Merkel 200 study. ESMO Open. (2024) 9:103461. doi: 10.1016/j.esmoop.2024.103461, PMID: 38744102 PMC11108812

[4] Gershenwald JE Scolyer RA . Melanoma staging: american joint committee on cancer (AJCC) 8th edition and beyond. Ann Surg Oncol. (2018) 25:2105–10. doi: 10.1245/s10434-018-6513-7, PMID: 29850954

[5] Kirkwood JM Del Vecchio M Weber J Hoeller C Grob JJ Mohr P . Adjuvant nivolumab in resected stage IIB/C melanoma: primary results from the randomized, phase 3 CheckMate 76K trial. Nat Med. (2023) 29:2835–43. doi: 10.1038/s41591-023-02583-2, PMID: 37845511 PMC10667090

[6] Vasudevan SS Patel T DiGiovanni J Nathan CO Khandelwal AR . Current efficacy and safety and survival outcomes of cemiplimab in advanced cutaneous squamous cell carcinoma: A systematic review and meta-analysis. J Invest Dermatol. (2025) 145(10):2623––2626.e5. doi: 10.1016/j.jid.2025.03.016, PMID: 40180032

[7] Stratigos AJ Sekulic A Peris K Bechter O Prey S Kaatz M . Cemiplimab in locally advanced basal cell carcinoma after hedgehog inhibitor therapy: an open-label, multi-centre, single-arm, phase 2 trial. Lancet Oncol. (2021) 22:848–57. doi: 10.1016/S1470-2045(21)00126-1, PMID: 34000246

[8] Sharma P Allison JP . Dissecting the mechanisms of immune checkpoint therapy. Nat Rev Immunol. (2020) 20:75–6. doi: 10.1038/s41577-020-0275-8, PMID: 31925406

[9] Hassel JC Heinzerling L Aberle J Bähr O Eigentler TK Grimm MO . Combined immune checkpoint blockade (anti-PD-1/anti-CTLA-4): Evaluation and management of adverse drug reactions. Cancer Treat Rev. (2017) 57:36–49. doi: 10.1016/j.ctrv.2017.05.003, PMID: 28550712

[10] Donia M Jespersen H Jalving M Lee R Eriksson H Hoeller C . Adjuvant immunotherapy in the modern management of resectable melanoma: current status and outlook to 2028. ESMO Open. (2025) 10:104295. doi: 10.1016/j.esmoop.2025.104295, PMID: 39954389 PMC11872484

[11] Gross ND Miller DM Khushalani NI Divi V Ruiz ES Lipson EJ . Neoadjuvant cemiplimab and surgery for stage II-IV cutaneous squamous-cell carcinoma: follow-up and survival outcomes of a single-arm, multicentre, phase 2 study. Lancet Oncol. (2023) 24:1196–205. doi: 10.1016/S1470-2045(23)00459-X, PMID: 37875144

[12] Patel SP Othus M Chen Y Wright GP Jr. Yost KJ Hyngstrom JR . Neoadjuvant-adjuvant or adjuvant-only pembrolizumab in advanced melanoma. N Engl J Med. (2023) 388:813–23. doi: 10.1056/NEJMoa2211437, PMID: 36856617 PMC10410527

[13] Blank CU Lucas MW Scolyer RA Van De Wiel BA Menzies AM Lopez-Yurda M . Neoadjuvant nivolumab and ipilimumab in resectable stage III melanoma. N Engl J Med. (2024) 391:1696–708. doi: 10.1056/NEJMoa2402604, PMID: 38828984

[14] Postow MA Sidlow R Hellmann MD . Immune-related adverse events associated with immune checkpoint blockade. N Engl J Med. (2018) 378:158–68. doi: 10.1056/NEJMra1703481, PMID: 29320654

[15] Pellegrini M Calzascia T Toe JG Preston SP Lin AE Elford AR . IL-7 engages multiple mechanisms to overcome chronic viral infection and limit organ pathology. Cell. (2011) 144:601–13. doi: 10.1016/j.cell.2011.01.011, PMID: 21295337

[16] Tokunaga R Naseem M Lo JH Battaglin F Soni S Puccini A . B cell and B cell-related pathways for novel cancer treatments. Cancer Treat Rev. (2019) 73:10–9. doi: 10.1016/j.ctrv.2018.12.001, PMID: 30551036 PMC7505165

[17] Reschke R Shapiro JW Yu J Rouhani SJ Olson DJ Zha Y . Checkpoint blockade-induced dermatitis and colitis are dominated by tissue-resident memory T cells and Th1/Tc1 cytokines. Cancer Immunol Res. (2022) 10:1167–74. doi: 10.1158/2326-6066.CIR-22-0362, PMID: 35977003 PMC9530647

[18] Dimitriou F Cheng PF Saltari A Schaper-Gerhardt K Staeger R Haunerdinger V . A targetable type III immune response with increase of IL-17A expressing CD4(+) T cells is associated with immunotherapy-induced toxicity in melanoma. Nat Cancer. (2024) 5:1390–408. doi: 10.1038/s43018-024-00810-4, PMID: 39210005 PMC11424476

[19] Nuñez NG Berner F Friebel E Unger S Wyss N Gomez JM . Immune signatures predict development of autoimmune toxicity in patients with cancer treated with immune checkpoint inhibitors. Med. (2023) 4:113–29.e7. doi: 10.1016/j.medj.2022.12.007, PMID: 36693381

[20] Lim SY Lee JH Gide TN Menzies AM Guminski A Carlino MS . Circulating cytokines predict immune-related toxicity in melanoma patients receiving anti-PD-1-based immunotherapy. Clin Cancer Res. (2019) 25:1557–63. doi: 10.1158/1078-0432.CCR-18-2795, PMID: 30409824

[21] Taylor CA Watson RA Tong O Ye W Nassiri I Gilchrist JJ . IL7 genetic variation and toxicity to immune checkpoint blockade in patients with melanoma. Nat Med. (2022) 28:2592–600. doi: 10.1038/s41591-022-02095-5, PMID: 36526722 PMC9800275

[22] Açar FP Acar C Gunenc D Arisoy Ç Ece Solmaz A Gecgel A . Predicting immune-related adverse events in patients with melanoma: the role of interleukin-7 rs16906115 polymorphism and lymphocyte dynamics. Front Immunol. (2025) 16:1616325. doi: 10.3389/fimmu.2025.1616325, PMID: 40642097 PMC12240767

[23] Bouloudani T Pupier G Sautès-Fridman C Fridman WH . B cells are major players in cancer immunity. Immunol Lett. (2025) 276:107064. doi: 10.1016/j.imlet.2025.107064, PMID: 40749982

[24] Zeng Z Mao H Lei Q He Y . IL-7 in autoimmune diseases: mechanisms and therapeutic potential. Front Immunol. (2025) 16:1545760. doi: 10.3389/fimmu.2025.1545760, PMID: 40313966 PMC12043607

[25] Wulfken LM Becker JC Hayajneh R Wagner AD Schaper-Gerhardt K Flatt N . Case report: sustained remission due to PD-1-inhibition in a metastatic melanoma patient with depleted B cells. Front Immunol. (2021) 12:733961. doi: 10.3389/fimmu.2021.733961, PMID: 34675925 PMC8525286

[26] Khoja L Atenafu EG Templeton A Qye Y Chappell MA Saibil S . The full blood count as a biomarker of outcome and toxicity in ipilimumab-treated cutaneous metastatic melanoma. Cancer Med. (2016) 5:2792–9. doi: 10.1002/cam4.878, PMID: 27683208 PMC5083732

