# Supplementary material for: A composite score of serum cytokines enables early identification of patients at high risk for irAEs under immune checkpoint inhibition
Source: Front Immunol. 2025 Nov 27;16:1733357. doi: 10.3389/fimmu.2025.1733357 (PMC12696155; doi:10.3389/fimmu.2025.1733357)
Supplement: Supplementary file 1 [file DataSheet1.docx]

**Supplementary Figures/Tables**

**Suppl. Figure 1:**

**
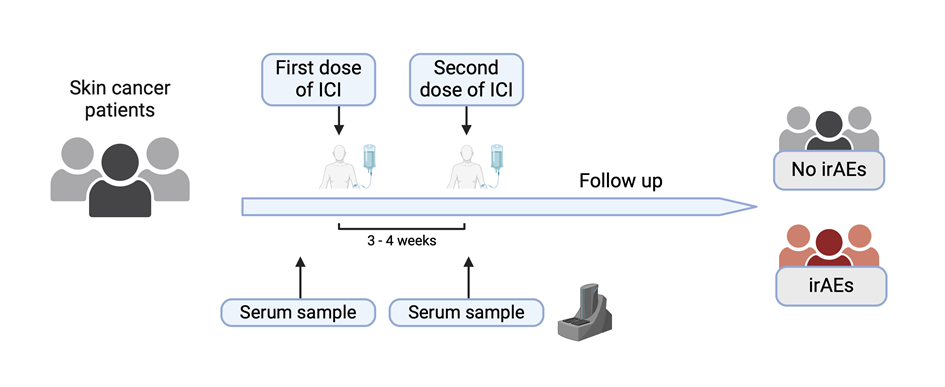
**

**Suppl. Figure 1:** **Study design**. Serum samples from skin cancer patients receiving immunotherapy were taken prior to ICI therapy and 3-4 weeks after the first treatment cycle. Patients were further prospectively monitored in regard of irAEs. Baseline protein level and dynamic changes after the first ICI treatment were analyzed and correlated with the occurrence of irAEs. ICI = immune checkpoint inhibition. IrAEs = immune-related adverse events.

Created with biorender.com

**Suppl. Figure 2**

**Suppl. Figure 2: Distribution of baseline parameters in patients with and without irAEs.** Violin plots showing baseline parameters in patients who developed irAEs compared to patients with no irAEs. Comparison between groups were performed using Mann-Whitney test. *p < 0.05, **p < 0.01, ***p <0.001: AE= adverse event, Tox= toxicity, NLR= neutrophil to lymphocyte ratio; derived neutrophil to lymphocytes ratio. LDH = Lactate dehydrogenase

**Suppl. Table 1** Univariable and multivariable logistic regression analysis of patients with and without severe irAEs (parameter above the line were included) of different baseline and dynamic parameters. OR = odds ratio, CI = confidence interval, irAEs = immune related adverse events

|  | **Parameter** | **N** | **Univariable (p; OR 95% CI)** | *p-value* | **Multivariable (p; OR 95% CI)** | *p-value* |
| --- | --- | --- | --- | --- | --- | --- |
| **irAE (grade3-4)** | **Therapy (Ref.: mICI)** | **80** | **15,000 (3,855-58,366)** | **< 0,001** | **11,170 (2,201-56,675)** | **0,004** |
|  | Age | 80 | 0,951 (0,917-0,986) | 0,007 | 0,978 (0,937-1,020) | 0,301 |
|  | IL-7 (Log2FC) | 80 | 0,338 (0,144-0,794) | 0,013 | 0,410 (0,156-1,077) | 0,07 |
|  | IL1RA (Log2FC) | 80 | 2,085 (1,138-3,821) | 0,017 | 0,969 (0,339-2,774) | 0,954 |
|  | IFNy (Log2FC) | 80 | 1,426 (1,061-1,916) | 0,018 | 1,120 (0,722-1,739) | 0,613 |
|  | BCA/CXCL13 (Log2FC) | 75 | 2,664 (1,156-6,136) | 0,021 | 1,007 (0,308-3,297) | 0,99 |
|  | BRAF (Ref.: WT) | 71 | 3,125 (1128-8,657) | 0,028 |  |  |
|  | TSLP (Log2FC) | 75 | 2,299 (1,061-4,982) | 0,035 |  |  |
|  | Eotaxin-3 (Log2FC) | 77 | 2,1884 (1,00-4,750) | 0,049 |  |  |
|  | IL-27 (Log2FC) | 80 | 1,864 (1,002-3,470) | 0,049 |  |  |
|  | MIP-1a (Log2FC) | 79 | 1,435 (0,770-2,671) | 0,255 |  |  |
|  | CRP (Log2FC) | 80 | 1,014 (0,915-1,123) | 0,791 |  |  |

**Suppl. Table 2:** Univariate logistic regression analysis of patients with and without specific irAE. OR = odds ratio, CI = confidence interval, irAE = immune related adverse events

|  | **Parameter** | **N** | **Univariable (p; OR 95% CI)** | ***p-value*** |
| --- | --- | --- | --- | --- |
| Cytokine risk score | AE (grade 1-4) | 119 | 2,719 (1,559-4,740) | **<0,001** |
|  | AE (grade 3-4) | 74 | 2,577 (1,417-4,687) | **0,002** |
|  | AE_skin | 60 | 2,601 (1,301-5,201) | **0,007** |
|  | AE_colon | 54 | 2,752 (1,32-5,735) | **0,007** |
|  | AE_pituitary gland | 56 | 1,465 (0,601-3,573) | 0,401 |
|  | AE_thyroid | 55 | 3,846 (1,652-8,954) | **0,002** |
|  | AE_liver | 56 | 6,705 (2,089-21,514) | **0,001** |

A B


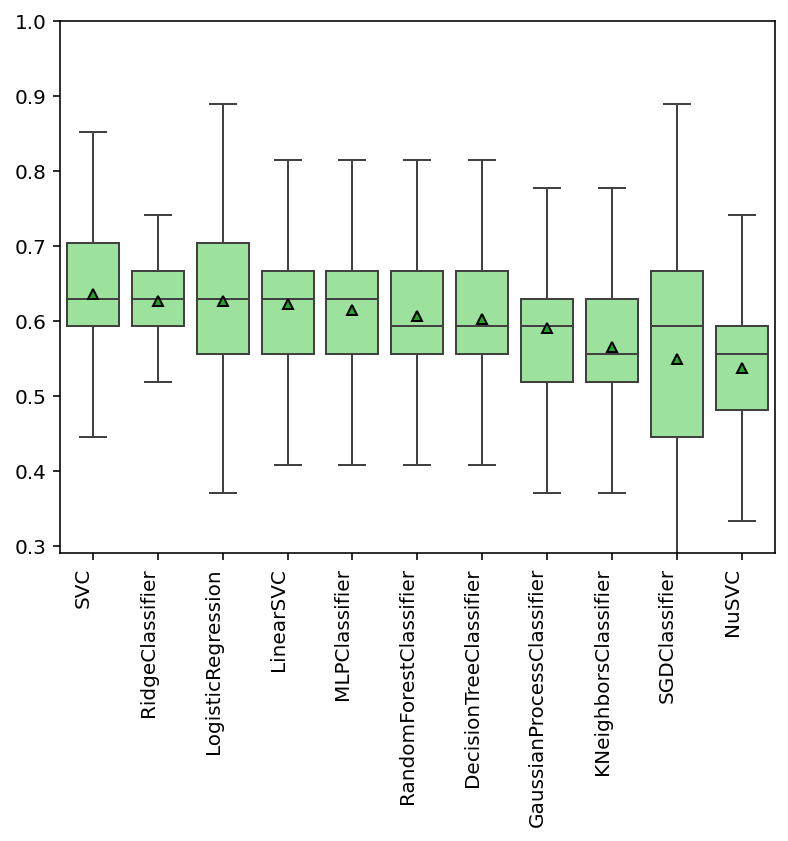
**
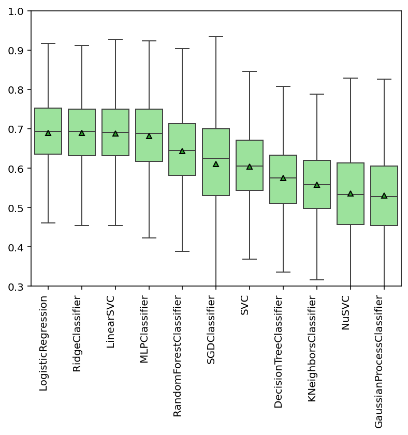
**

**Suppl. Figure 3: Boxplot of A) AUCs and B) overall classification accuracy from different models tested.**Models were trained on the parameters age, therapy type, IL-7LOG2FC, BCALog2FC and IL1RALog2FC, with random 80/20 training/test splits. The selection of parameters is identical to our proposed cytokine risk score. Each model was trained and evaluated 1000 times on independently resampled splits. The distribution of AUC values across repetitions is shown for each classifier. The boxes indicate the interquartile range (IQR), whiskers represent the 1.5×IQR, and black triangles denote the mean AUC. The evaluated classifiers represent a broad range of common machine learning algorithms implemented in *scikit-learn*:

**SVC / LinearSVC / NuSVC:** Support Vector Classifiers with different optimization strategies and regularization schemes.

- **RidgeClassifier / LogisticRegression:** Linear models with L2 regularization, providing interpretable baselines.
- **MLPClassifier:** A feed-forward neural network (multi-layer perceptron) capable of learning nonlinear relationships.
- **RandomForestClassifier / DecisionTreeClassifier:** Tree-based ensemble and single-tree methods capturing nonlinear feature interactions.
- **GaussianProcessClassifier:** A nonparametric Bayesian model that estimates class probabilities using Gaussian processes.
- **KNeighborsClassifier:** A distance-based algorithm that classifies samples based on nearest neighbors in feature space.
- **SGDClassifier:** A linear classifier trained using stochastic gradient descent, suitable for large-scale data.

Overall, **logistic regression** demonstrated consistently strong performance, ranking among the top models in terms of AUC and achieving the highest mean accuracy. This finding further substantiates the rationale underlying the proposed cytokine score, suggesting that the discriminative signal captured by the selected features can be effectively modeled using a linear approach.

*A B*

***
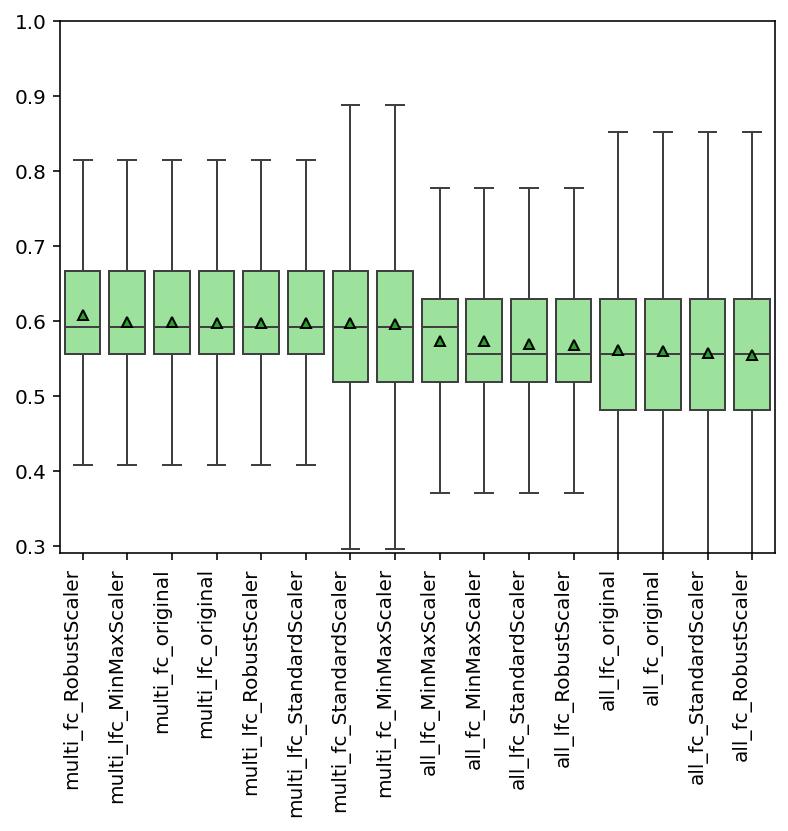

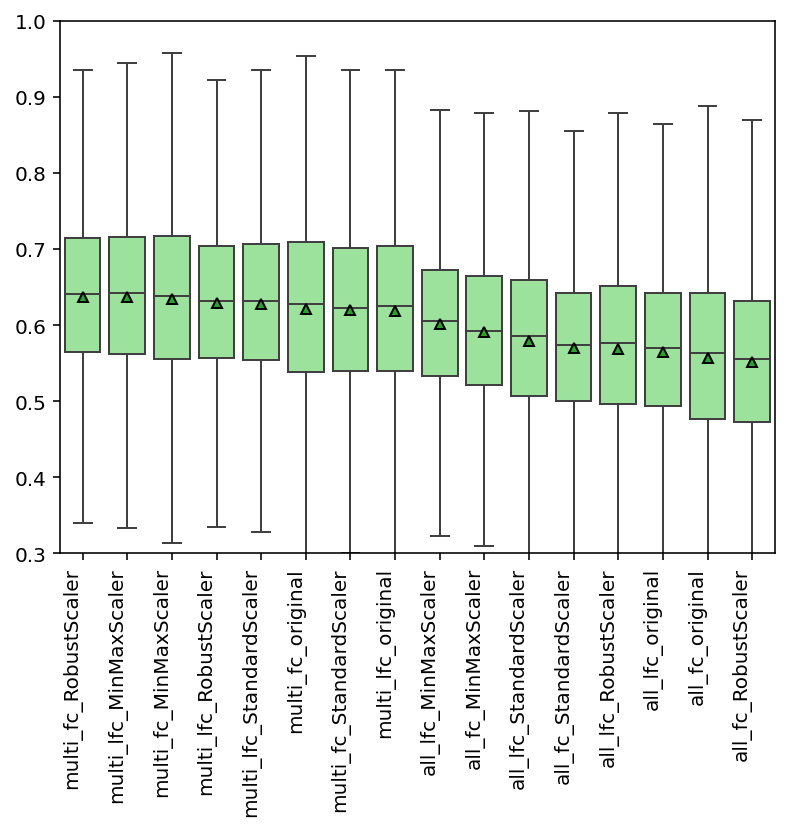
***

**Suppl. Figure 4: Boxplot of A) AUCs and B) overall classification accuracy from different settings tested.**

We define setting as a distinct way of data preparation that is encoded in the setting’s name. Each setting’s name consists of the three components “{filtering}_{preprocessing}_{scaling}”. See the last paragraph for details on each component.

In suppl. figure 3, different machine learning models were trained and tested on the subset of significant cytokines and subsequently compared with regard to their accuracy and AUC. This process was repeated, but instead of different models, we now compared different seetings. For clarity, the results of suppl. figure 3 are all represented in the setting multi_lfc_original in this figure.

As a result, models perform better on selected cytokines (“multi”) rather than the full dataset (“all”) which supports the idea of the presented cytokine risk score. Other differences among the settings yields no significant difference.

Definition of a setting “{filtering}_{preprocessing}_{scaling}”:

**filtering**:

**all:** age, therapy_num and all 40 cytokines are available to the models.

**multi:** age, therapy_num and IL_7_LOG2FC, BCA_1_BCL_LOG2FC, IL_1RA_LOG2FC are available to the models.

**preprocessing**:

**lfc:** log2 fold changes are used for the cytokine ratios

**fc:** fold changes are used for the cytokine ratios

**scaling**:

**no scaling:** Values are not scaled.

**MinMax scaling**: Within a column, values are linearly transformed to the interval [0,1]. The largest value is transformed to 1 and the smallest value is transformed to 0.

**z score normalization:** Within a column, values are centered around the mean and divided by the column’s standard deviation deviation

**Robust scaling**: Within a column, values are centered around the median and divided by the interquantile range. The interquantile range is the difference between the 25% and 75% quantile.
